# Equivalent conditions and applications of a class of Hilbert-type integral inequalities involving multiple functions with quasi-homogeneous kernels

**DOI:** 10.1186/s13660-018-1797-5

**Published:** 2018-08-09

**Authors:** Junfei Cao, Bing He, Yong Hong, Bicheng Yang

**Affiliations:** 1 Department of Mathematics, Guangdong University of Education, Guangzhou, P.R. China; 2 College of Mathematics and Statistics, Guangdong University of Finance and Economics, Guangzhou, P.R. China

**Keywords:** 26D15, 47A07, Hilbert-type integral inequality, Quasi-homogeneous kernel, Equivalent conditions, Best constant factor

## Abstract

Let $K(x_{1},\ldots,x_{n})$ satisfy

$$K(x_{1},\ldots,tx_{i},\ldots,x_{n})=t^{\lambda\lambda _{i}}K \bigl(t^{-\frac{\lambda_{i}}{\lambda_{1}}}x_{1},\ldots,x_{i}, \ldots,t^{-\frac {\lambda _{i}}{\lambda_{n}}}x_{n} \bigr) $$

for $t>0$. With this integral kernel, by using the method and technique of weight coefficients, the equivalent conditions and the best constant factors for the validity of Hilbert-type integral inequalities involving multiple functions are discussed. Finally, the applications of the integral inequalities are considered.

## Introduction

Let $x=(x_{1},\ldots,x_{n}), \mathbf{R}_{+}^{n}=\{x=(x_{1},\ldots ,x_{n}):x_{i}>0\ (i=1,\ldots,n)\},r>1,f(t)\geq0$, and *α* be a constant. Set

$$L_{\alpha}^{r}(0,+\infty)= \biggl\{ f(t): \Vert f \Vert _{r,\alpha}= \biggl( \int_{0}^{+\infty}t^{\alpha}f^{r}(t)\,dt \biggr) ^{1/r}< +\infty \biggr\} . $$

If $\sum_{i=1}^{n}\frac{1}{p_{i}}=1\ (p_{i}>1,i=1,\ldots,n),\alpha _{i}\in \mathbf{R},f_{i}(x_{i})\in L_{\alpha_{i}}^{p_{i}}(0,+\infty)$ $(i=1,\ldots ,n),K(x_{1},\ldots,x_{n})\geq0$, *M* is a constant, then we name the following inequality a Hilbert-type integral inequality:

$$\int_{\mathbf{R}_{+}^{n}}K(x_{1},\ldots,x_{n})\prod _{i=1}^{n}f_{i}(x_{i})\,dx_{1} \cdots \,dx_{n}\leq M\prod_{i=1}^{n} \Vert f_{i} \Vert _{p_{i},\alpha_{i}}. $$

An integral kernel $K(x_{1},\ldots,x_{n})$ is said to be a quasi-homogeneous function with parameters $(\lambda,\lambda _{1},\ldots ,\lambda_{n})$ if, for $t>0$,

$$K(x_{1},\ldots,tx_{i},\ldots,x_{n})=t^{\lambda\lambda _{i}}K \bigl(t^{-\frac{\lambda_{i}}{\lambda_{1}}}x_{1},\ldots,x_{i}, \ldots,t^{-\frac {\lambda _{i}}{\lambda_{n}}}x_{n} \bigr) \quad (i=1,\ldots,n). $$

Obviously, $K(x_{1},\ldots,x_{n})$ becomes a homogeneous function of order $\lambda\lambda_{0}$ when $\lambda_{1}=\lambda_{2}=\cdots=\lambda _{n}=\lambda_{0}$.

So far, many good results have been obtained in the study of Hilbert-type inequalities (cf. [1–24]). What are the necessary and sufficient conditions for the validity of a Hilbert-type inequality? What is the best constant factor when the inequality holds? The research on such problems is undoubtedly of great significance to the study and applications of Hilbert-type inequality theory, but unfortunately, the research on this type of problems is rarely seen.

In this paper, we focus on the quasi-homogeneous integral kernels, discuss the equivalent conditions for the validity of Hilbert-type integral inequalities involving multiple functions, and obtain the expressions of the best constant factors when the inequalities are established. Finally, we discuss their applications.

## Some lemmas

### Lemma 1

*Let integer* $n\geq2,\sum_{i=1}^{n}\frac{1}{p_{i}}=1\ (p_{i}>1,i=1,\ldots,n),\lambda\in\mathbf{R},\alpha_{i}\in\mathbf {R},\lambda_{i}>0$ (*or* $\lambda_{i}<0$) $(i=1,\ldots,n)$, *and* $K(x_{1},\ldots,x_{n})$ *be a nonnegative measurable function with parameters* $(\lambda,\lambda_{1},\ldots,\lambda_{n}),\sum_{i=1}^{n}\frac{\alpha _{i}+1}{\lambda_{i}p_{i}}=\lambda+\sum_{i=1}^{n}\frac{1}{\lambda_{i}}$. *Set*

$$\begin{aligned} W_{j} ={}& \int_{\mathbf{R}_{+}^{n-1}}K(u_{1},\ldots,u_{j-1},1,u_{j+1}, \ldots,u_{n}) \\ &{}\times\prod_{i=1(i\neq j)}^{n}u_{i}^{-\frac{\alpha_{i}+1}{p_{i}}}\,du_{1} \cdots \,du_{j-1}\,du_{j+1}\cdots \,du_{n}. \end{aligned}$$

*Then* $\frac{1}{\lambda_{1}}W_{1}=\frac{1}{\lambda_{2}}W_{2}=\cdots =\frac{1}{\lambda n}W_{n}$, *and*

$$\begin{aligned} \omega_{j}(x_{j})& = \int_{\mathbf{R}_{+}^{n-1}}K(x_{1},\ldots,x_{n})\prod _{i=1(i\neq j)}^{n}x_{i}^{-\frac{\alpha_{i}+1}{p_{i}}}\,dx_{1} \cdots \,dx_{j-1}\,dx_{j+1}\cdots \,dx_{n} \\ &= x_{j}^{\lambda_{j} ( \lambda+\sum_{i=1(i\neq j)}^{n}\frac {\alpha _{i}+1}{\lambda_{i}p_{i}}+\sum_{i=1(i\neq j)}^{n}\frac{1}{\lambda_{i}} ) }W_{j}, \end{aligned}$$

*where* $j=1,\ldots,n$.

### Proof

When $j\geq2$, by $\sum_{i=1}^{n}\frac{\alpha_{i}+1}{\lambda_{i}p_{i}}=\lambda+\sum_{i=1}^{n}\frac{1}{\lambda_{i}}$,

$$\begin{aligned} W_{j} ={}& \int_{\mathbf{R}_{+}^{n-1}}u_{1}^{\lambda\lambda_{1}}K \biggl( 1,\frac{u_{2}}{u_{1}^{\frac{\lambda_{1}}{\lambda_{2}}}},\ldots,\frac {u_{j-1}}{u_{1}^{\frac{\lambda_{1}}{\lambda_{j-1}}}},\frac{1}{u_{1}^{\frac {\lambda _{1}}{\lambda_{j}}}}, \frac{u_{j+1}}{u_{1}^{\frac{\lambda_{1}}{\lambda _{j+1}}}},\ldots,\frac{u_{n}}{u_{1}^{\frac{\lambda_{1}}{\lambda _{n}}}} \biggr) \\ &{}\times\prod_{i=1(i\neq j)}^{n}u_{i}^{-\frac{\alpha_{i}+1}{p_{i}}}\,du_{1} \cdots \,du_{j-1}\,du_{j+1}\cdots \,du_{n} \\ ={}& \frac{\lambda_{j}}{\lambda_{1}} \int_{\mathbf{R}_{+}^{n-1}}K ( 1,t_{2},\ldots,t_{n} ) t_{j}^{\lambda_{j} ( -\lambda -\sum_{i=1}^{n}\frac{1}{\lambda_{i}}+\sum_{i=1(i\neq j)}^{n}\frac {\alpha _{i}+1}{\lambda_{i}p_{i}} ) } \\ &{}\times\prod_{i=2(i\neq j)}^{n}t_{i}^{-\frac{\alpha_{i}+1}{p_{i}}}\,dt_{2} \cdots \,dt_{n} \\ ={}& \frac{\lambda_{j}}{\lambda_{1}} \int_{\mathbf{R}_{+}^{n-1}}K ( 1,t_{2},\ldots,t_{n} ) t_{j}^{\lambda_{j} ( -\frac{\alpha _{j}+1}{\lambda_{j}p_{j}} ) }\prod_{i=2(i\neq j)}^{n}t_{i}^{-\frac{\alpha _{i}+1}{p_{i}}}\,dt_{2} \cdots \,dt_{n} \\ ={}& \frac{\lambda_{j}}{\lambda_{1}} \int_{\mathbf{R}_{+}^{n-1}}K ( 1,t_{2},\ldots,t_{n} ) \prod_{i=2}^{n}t_{i}^{-\frac{\alpha _{i}+1}{p_{i}}}\,dt_{2} \cdots \,dt_{n}=\frac{\lambda_{j}}{\lambda_{1}}W_{1}. \end{aligned}$$

Therefore $\frac{1}{\lambda_{j}}W_{j}=\frac{1}{\lambda _{1}}W_{1}\ (j\geq2)$. When $j=1,\ldots,n$, we also get

$$\begin{aligned} \omega_{j}(x_{j}) ={}& \int_{\mathbf{R}_{+}^{n-1}}x_{j}^{\lambda\lambda _{j}}K \biggl( \frac{x_{1}}{x_{j}^{\frac{\lambda_{j}}{\lambda 1}}},\ldots,\frac{x_{j-1}}{x_{j}^{\frac{\lambda_{j}}{\lambda_{j-1}}}},1,\frac {x_{j+1}}{x_{j}^{\frac{\lambda_{j}}{\lambda_{j+1}}}}, \ldots,\frac {x_{n}}{x_{j}^{\frac{\lambda_{j}}{\lambda_{n}}}} \biggr) \\ &{}\times\prod_{i=1(i\neq j)}^{n}x_{i}^{-\frac{\alpha_{i}+1}{p_{i}}}\,dx_{1} \cdots \,dx_{j-1}\,dx_{j+1}\cdots \,dx_{n} \\ ={}& x_{j}^{\lambda\lambda_{j}-\lambda_{j}\sum_{i=1(i\neq j)}^{n}\frac{ \alpha_{i}+1}{\lambda_{i}p_{i}}+\lambda_{j}\sum_{i=1(i\neq j)}^{n}\frac{1}{\lambda_{i}}} \\ &{}\times \int_{\mathbf{R}_{+}^{n-1}}K ( u_{1},\ldots,u_{j-1},1,u_{j+1} ,\ldots,u_{n} ) \\ &{}\times\prod_{i=1(i\neq j)}^{n}u_{i}^{-\frac{\alpha_{i}+1}{p_{i}}}\,du_{1} \cdots \,du_{j-1}\,du_{j+1}\cdots \,du_{n} \\ ={}& x_{j}^{\lambda_{j} ( \lambda+\sum_{i=1(i\neq j)}^{n}\frac {\alpha _{i}+1}{\lambda_{i}p_{i}}+\sum_{i=1(i\neq j)}^{n}\frac{1}{\lambda_{i}} ) }W_{j}. \end{aligned}$$

 □

### Lemma 2

([25])

*Let* $p_{i}>0,a_{i}>0,\alpha_{i}>0$ $(i=1,\ldots,n),\psi(u)$ *be measurable*. *Then*

$$\begin{aligned} &\int\cdots \int_{x_{i}>0, ( \frac{x_{1}}{a_{1}} ) ^{\alpha _{1}}+\cdots+( \frac{x_{n}}{a_{n}} ) ^{\alpha_{n}}\leq1}\psi \biggl( \biggl( \frac{x_{1}}{a_{1}} \biggr) ^{\alpha_{1}}+\cdots+ \biggl( \frac{x_{n}}{a_{n}} \biggr) ^{\alpha_{n}} \biggr) \\ &\qquad{}\times x_{1}^{p_{1}-1}\cdots x_{n}^{p_{n}-1}\,dx_{1} \cdots \,dx_{n} \\ &\quad = \frac{a_{1}^{p_{1}}\cdots a_{n}^{p_{n}}\Gamma( \frac {p_{1}}{\alpha _{1}} ) \cdots\Gamma( \frac{p_{n}}{\alpha n} ) }{\alpha _{1}\cdots\alpha_{n}\Gamma( \frac{p_{1}}{\alpha_{1}}+\cdots +\frac{p_{n}}{\alpha n} ) } \int_{0}^{1}\psi(t)t^{\frac{p_{1}}{\alpha _{1}}+\cdots+\frac{p_{n}}{\alpha n}-1}\,dt, \end{aligned}$$

*where* Γ *represents the gamma function*.

## Main results and their proofs

### Theorem 1

*Suppose that* $n\geq2,\sum_{i=1}^{n}\frac{1}{p_{i}}=1\ (p_{i}>1),\lambda\in\mathbf{R},\lambda_{i}>0$ (*or* $\lambda _{i}<0$), $\alpha_{i}\in\mathbf{R}$ $(i=1,\ldots,n)$. *If* $K(x_{1},\ldots ,x_{n})$ *is a quasi*-*homogeneous positive function with parameters* ($\lambda ,\lambda_{1},\ldots,\lambda_{n}$), *and*

$$W_{1}= \int_{\mathbf{R}_{+}^{n-1}}K(1,u_{2},\ldots,u_{n})\prod _{i=2}^{n}u_{i}^{-\frac{\alpha_{i}+1}{p_{i}}}\,du_{2} \cdots \,du_{n} $$

*is convergent*, *then*

(i) *the inequality*
  1 $$ \int_{\mathbf{R}_{+}^{n}}K(x_{1},\ldots,x_{n})\prod _{i=1}^{n}f_{i}(x_{i})\,dx_{1} \cdots \,dx_{n}\leq M\prod_{i=1}^{n} \Vert f_{i} \Vert _{p_{i},\alpha_{i}} $$
  *holds for some constant* $M>0$ *if and only if* $\sum_{i=1}^{n}\frac{\alpha _{i}+1}{\lambda_{i}p_{i}}=\lambda+\sum_{i=1}^{n}\frac{1}{\lambda_{i}}$, *where* $f_{i}(x_{i})\in L_{\alpha_{i}}^{p_{i}}(0,+\infty)$ $(i=1,\ldots ,n)$;
(ii) *if* (1) *holds*, *then its best constant factor is* $\inf M=\frac {W_{1}}{|\lambda_{1}|}\prod_{i=1}^{n}|\lambda_{i}|^{1/p_{i}}$.

### Proof

(i) Sufficiency. Assume that $\sum_{i=1}^{n}\frac{\alpha _{i}+1}{\lambda_{i}p_{i}}=\lambda+\sum_{i=1}^{n}\frac{1}{\lambda_{i}}$. Since

$$\prod_{j=1}^{n}x_{j}^{\frac{\alpha_{j}+1}{p_{j}}} \Biggl( \prod_{i=1}^{n}x_{i}^{-\frac{\alpha_{i}+1}{p_{i}}} \Biggr) ^{1/p_{j}}=\prod_{j=1}^{n}x_{j}^{\frac{\alpha_{j}+1}{p_{j}}} \prod_{i=1}^{n}x_{i}^{-\frac{\alpha_{i}+1}{p_{i}}\sum_{k=1}^{n}\frac {1}{p_{k}}}=1, $$

by Hölder’s inequality and Lemma 1, we obtain

$$\begin{aligned} &\int_{\mathbf{R}_{+}^{n}}K(x_{1},\ldots,x_{n})\prod _{i=1}^{n}f_{i}(x_{i})\,dx_{1} \cdots \,dx_{n} \\ &\quad= \int_{\mathbf{R}_{+}^{n}}K(x_{1},\ldots,x_{n}) \Biggl[ \prod_{j=1}^{n}x_{j}^{\frac{\alpha_{j}+1}{p_{j}}} \Biggl( \prod_{i=1}^{n}x_{i}^{-\frac{\alpha_{i}+1}{p_{i}}} \Biggr) ^{1/p_{j}}f_{j}(x_{j}) \Biggr] \,dx_{1}\cdots \,dx_{n} \\ &\quad \leq\prod_{j=1}^{n} \Biggl[ \int_{\mathbf{R}_{+}^{n}}x_{j}^{\alpha _{j}+1} \Biggl( \prod _{i=1}^{n}x_{i}^{-\frac{\alpha_{i}+1}{p_{i}}} \Biggr) f_{j}^{p_{j}}(x_{j})K(x_{1}, \ldots,x_{n})\,dx_{1}\cdots \,dx_{n} \Biggr] ^{1/p_{j}} \\ &\quad= \prod_{j=1}^{n} \Biggl[ \int_{0}^{+\infty}x_{j}^{\alpha_{j}+1-\frac{\alpha_{j}+1}{p_{j}}}f_{j}^{p_{j}}(x_{j}) \\ &\qquad{}\times \Biggl( \int_{\mathbf{R}_{+}^{n-1}}K(x_{1},\ldots,x_{n})\prod _{i=1(i\neq j)}^{n}x_{i}^{-\frac{\alpha_{i}+1}{p_{i}}}\,dx_{1} \cdots \,dx_{j-1}\,dx_{j+1}\cdots \,dx_{n} \Biggr) \,dx_{j} \Biggr] ^{1/p_{j}} \\ &\quad= \prod_{j=1}^{n} \biggl( \int_{0}^{+\infty}x_{j}^{\alpha_{j}+1-\frac{\alpha_{j}+1}{p_{j}}}f_{j}^{p_{j}}(x_{j}) \omega_{j}(x_{j})\,dx_{j} \biggr) ^{1/p_{j}} \\ &\quad= \prod_{j=1}^{n} \biggl[ \int_{0}^{+\infty}x_{j}^{\alpha_{j}+1-\frac {\alpha _{j}+1}{p_{j}}+\lambda_{j} ( \lambda-\sum_{i=1(i\neq j)}^{n}\frac{ \alpha_{i}+1}{\lambda_{i}p_{i}}+\sum_{i=1(i\neq j)}^{n}\frac {1}{\lambda _{i}} ) }f_{j}^{p_{j}}(x_{j})W_{j}\,dx_{j} \biggr]^{1/p_{j}} \\ &\quad= \prod_{j=1}^{n}W_{j}^{1/p_{j}} \prod_{j=1}^{n} \biggl[ \int_{0}^{+\infty }x_{j}^{\lambda_{j} ( \frac{\alpha_{j}}{\lambda_{j}}-\sum _{i=1}^{n}\frac{\alpha_{i}+1}{\lambda_{i}p_{i}}+\lambda+\sum_{i=1}^{n}\frac{1}{ \lambda_{i}} ) }f_{j}^{p_{j}}(x_{j})\,dx_{j} \biggr]^{1/p_{j}} \\ &\quad= \prod_{i=1}^{n}W_{i}^{1/p_{i}} \prod_{i=1}^{n} \biggl( \int_{0}^{+\infty }x_{i}^{\alpha_{i}}f_{i}^{p_{i}}(x_{i})\,dx_{i} \biggr) ^{1/p_{i}} \\ &\quad = \frac{W_{1}}{ \vert \lambda_{1} \vert } \Biggl( \prod_{i=1}^{n} \vert \lambda_{i} \vert ^{1/p_{i}} \Biggr) \prod _{i=1}^{n} \Vert f_{i} \Vert _{p_{i},\alpha_{i}}, \end{aligned}$$

thus (1) holds when taking any constant $M\geq\frac {W_{1}}{|\lambda _{1}|}\prod_{i=1}^{n}|\lambda_{i}|^{1/p_{i}}$.

Necessity. Assume that (1) holds. Set $c=\sum_{i=1}^{n}\frac {\alpha _{i}+1}{\lambda_{i}p_{i}}-\lambda-\sum_{i=1}^{n}\frac{1}{\lambda_{i}}$. Next we will prove $c=0$.

First consider the case of $\lambda_{i}>0\ (i=1,\ldots,n)$. If $c>0$, for $0<\varepsilon<c$, take

$$f_{i}(x_{i})= \textstyle\begin{cases} x{}_{i}^{(-\alpha_{i}-1+\lambda_{i}\varepsilon)/p_{i}}, & 0< x_{i}\leq1, \\ 0, & x_{i}>1,\end{cases} $$

where $i=1,\ldots,n$. Then

2 $$\begin{aligned} &\prod_{i=1}^{n} \Vert f_{i} \Vert _{p_{i},\alpha_{i}}=\prod_{i=1}^{n} \biggl( \int_{0}^{1}x_{i}^{-1+\lambda_{i}\varepsilon} \, dx_{i} \biggr) ^{1/p_{i}}=\frac{1}{\varepsilon{}}\prod _{i=1}^{n} \biggl( \frac {1}{\lambda _{i}{}} \biggr) ^{1/p_{i}}, \end{aligned}$$

3 $$\begin{aligned} &\int_{\mathbf{R}_{+}^{n}}K(x_{1},\ldots,x_{n})\prod _{i=1}^{n}f_{i}(x_{i})\,dx_{1}\cdots\,dx_{n} \\ &\quad= \int_{0}^{1}x_{1}^{(-\alpha_{1}-1+\lambda_{1}\varepsilon)/p_{1}} \\ &\qquad{}\times \Biggl( \int_{0}^{1}\cdots \int_{0}^{1}K(x_{1},x_{2}, \ldots,x_{n})\prod_{i=2}^{n}x_{i}^{(-\alpha_{i}-1+\lambda _{i}\varepsilon )/p_{i}}\,dx_{2}\cdots\,dx_{n} \Biggr) \, dx_{1} \\ &\quad= \int_{0}^{1}x_{1}^{(-\alpha_{1}-1+\lambda_{1}\varepsilon )/p_{1}+\lambda\lambda_{1}} \Biggl( \int_{0}^{1}\cdots \int_{0}^{1}K \bigl(1,x_{1}^{-\frac{\lambda_{1}}{\lambda_{2}}}x_{2}, \ldots,x_{1}^{-\frac{\lambda_{1}}{\lambda n}}x_{n} \bigr) \\ &\qquad{}\times\prod_{i=2}^{n}x_{i}^{(-\alpha_{i}-1+\lambda _{i}\varepsilon)/p_{i}} \,dx_{2}\cdots\,dx_{n} \Biggr) \,{d}x_{1} \\ &\quad= \int_{0}^{1}x_{1}^{-\frac{\alpha_{1}+1-\lambda_{1}\varepsilon }{p_{1}}+\lambda\lambda_{1}+\lambda_{1}\sum_{i=2}^{n}\frac{1}{\lambda_{i}}-\lambda_{1}\sum_{i=2}^{n}\frac{\alpha_{i}+1-\lambda_{i}\varepsilon }{\lambda_{i}p_{i}}} \Biggl( \int_{0}^{x_{1}^{-\lambda_{1}/\lambda _{2}}}\cdots \int_{0}^{x_{n}^{-\lambda_{1}/\lambda_{n}}} \\ &\qquad{}\times K(1,u_{2},\ldots,u_{n})\prod _{i=2}^{n}u_{i}^{(-\alpha _{i}-1+\lambda_{i}\varepsilon)/p_{i}} \, du_{2}\cdots\,du_{n} \Biggr) \, dx_{1} \\ &\quad = \int_{0}^{1}x_{1}^{\lambda_{1} ( \lambda+\sum_{i=1}^{n}\frac {1}{\lambda_{i}}-\sum_{i=1}^{n}\frac{\alpha_{i}+1}{\lambda _{i}p_{i}}-\frac{1}{\lambda_{1}}+\varepsilon) } \Biggl( \int_{0}^{x_{1}^{-\lambda _{1}/\lambda_{2}}}\cdots \int_{0}^{x_{n}^{-\lambda_{1}/\lambda _{n}}} \\ &\qquad{}\times K(1,u_{2},\ldots,u_{n})\prod _{i=2}^{n}u_{i}^{(-\alpha _{i}-1+\lambda_{i}\varepsilon)/p_{i}} \, du_{2}\cdots\,du_{n} \Biggr) \, dx_{1} \\ &\quad\geq \int_{0}^{1}x_{1}^{-1-\lambda_{1}c+\lambda_{1}\varepsilon } \,{d}x_{1} \Biggl( \int_{0}^{1}\cdots \int_{0}^{1}K(1,u_{2},\ldots ,u_{n}) \\ &\qquad{}\times\prod_{i=2}^{n}u_{i}^{(-\alpha_{i}-1+\lambda _{i}\varepsilon)/p_{i}} \,du_{2}\cdots\,du_{n} \Biggr). \end{aligned}$$

It follows from (1), (2), and (3) that

4 $$\begin{aligned} &\int_{0}^{1}x_{1}^{-1-\lambda_{1}c+\lambda_{1}\varepsilon}\, {d}x_{1} \Biggl( \int_{0}^{1}\cdots \int_{0}^{1}K(1,u_{2},\ldots ,u_{n}) \prod_{i=2}^{n}u_{i}^{(-\alpha_{i}-1+\lambda _{i}\varepsilon)/p_{i}} \,du_{2}\cdots\,du_{n} \Biggr) \\ &\quad \leq\frac{M}{\varepsilon}\prod_{i=1}^{n} \biggl( \frac{1}{\lambda _{i}{}} \biggr) ^{1/p_{i}}. \end{aligned}$$

Since $-1-\lambda_{1}c+\lambda_{1}\varepsilon<-1$, $\int_{0}^{1}x_{1}^{-1-\lambda_{1}c+\lambda_{1}\varepsilon}\, {d}x_{1}$ diverges to +∞. Whence it is a contradiction to (4). In other words, it is not valid for $c>0$.

If $c<0$, for $0<\varepsilon<-c$, take

$$f_{i}(x_{i})= \textstyle\begin{cases} x{}_{i}^{(-\alpha_{i}-1-\lambda_{i}\varepsilon)/p_{i}}, & x_{i}\geq 1, \\ 0, & 0< x_{i}< 1,\end{cases} $$

where $i=1,\ldots,n$. Similarly, we get

5 $$\begin{aligned} &\int_{1}^{+\infty}x_{1}^{-1+\lambda_{1}c-\lambda_{1}\varepsilon}\,dx_{1} \\ &\qquad{}\times \Biggl( \int_{1}^{+\infty}\cdots \int_{1}^{+\infty }K(1,u_{2}, \ldots,u_{n})\prod_{i=2}^{n}u_{i}^{(-\alpha_{i}-1-\lambda _{i}\varepsilon)/p_{i}} \,du_{2}\cdots\,du_{n} \Biggr) \\ &\quad \leq\frac{M}{\varepsilon}\prod_{i=1}^{n} \biggl( \frac{1}{\lambda _{i}{}} \biggr) ^{1/p_{i}}. \end{aligned}$$

Since $-1-\lambda_{1}c+ \lambda_{1}\varepsilon>-1$ and $\int_{1}^{+\infty }x_{1}^{-1-\lambda_{1}c-\lambda_{1}\varepsilon} \,dx_{1}$ diverges to +∞, which contradicts the above inequality, hence it does not hold for $c<0$.

To sum up, we have $c=0$ for $\lambda_{i}>0\ ( i=1,\ldots,n ) $.

Now let us consider the case of $\lambda_{i}<0\ ( i=1,\ldots,n ) $. If $c>0$, for $0< \varepsilon<c$, take

$$f_{i}(x_{i})= \textstyle\begin{cases} x{}_{i}^{(-\alpha_{i}-1+\lambda_{i}\varepsilon)/p_{i}}, & x_{i}\geq 1, \\ 0, & 0< x_{i}< 1,\end{cases} $$

where $i=1,\ldots,n$. Consequently,

6 $$\begin{aligned} &\prod_{i=1}^{n} \Vert f_{i} \Vert _{p_{i},\alpha_{i}}=\prod_{i=1}^{n} \biggl( \int_{1}^{+\infty}x_{i}^{-1+\lambda_{i}\varepsilon}\, {d}x_{i} \biggr) ^{1/p_{i}}=\frac{1}{\varepsilon}\prod _{i=1}^{n} \biggl( \frac{1}{-\lambda _{i}{}} \biggr) ^{1/p{}_{i}}{}, \end{aligned}$$

7 $$\begin{aligned} &\int_{\mathbf{R}_{+}^{n}}K(x_{1},\ldots,x_{n})\prod _{i=1}^{n}f_{i}(x_{i})\,dx_{1}\cdots\,dx_{n} \\ &\quad= \int_{1}^{+\infty}x_{1}^{(-\alpha_{1}-1+\lambda_{1}\varepsilon )/p_{1}} \Biggl( \int_{1}^{+\infty}\cdots \int_{1}^{+\infty }x_{1}^{\lambda \lambda_{1}}K \bigl(1,x_{1}^{-\frac{\lambda_{1}}{\lambda_{2}}}x_{2},\ldots ,x_{1}^{-\frac{\lambda_{1}}{\lambda_{n}}}x_{n} \bigr) \\ &\qquad{}\times\prod_{i=2}^{n}x_{i}^{(-\alpha_{i}-1+\lambda _{i}\varepsilon)/p_{i}} \,dx_{2}\cdots\,dx_{n} \Biggr) \,{d}x_{1} \\ &\quad= \int_{1}^{+\infty}x_{1}^{-\frac{\alpha_{1}+1-\lambda _{1}\varepsilon}{p_{1}}+\lambda\lambda_{1}+\lambda_{1}\sum_{i=2}^{n}\frac{1}{\lambda _{i}}-\lambda_{1}\sum_{i=2}^{n}\frac{\alpha_{i}+1-\lambda_{i}\varepsilon }{\lambda_{i}p_{i}}} \Biggl( \int_{x_{1}^{-\lambda_{1}/\lambda _{2}}}^{+\infty }\cdots \int_{x_{n}^{-\lambda_{1}/\lambda_{n}}}^{+\infty} \\ &\qquad{}\times K(1,u_{2},\ldots,u_{n})\prod _{i=2}^{n}u_{i}^{(-\alpha _{i}-1+\lambda_{i}\varepsilon)/p_{i}} \, du_{2}\cdots\,du_{n} \Biggr) \, dx_{1} \\ &\quad\geq \int_{1}^{+\infty}x_{1}^{-1-\lambda_{1}c+\lambda _{1}\varepsilon}\,dx_{1} \Biggl( \int_{1}^{+\infty}\cdots \int_{1}^{+\infty }K(1,u_{2}, \ldots,u_{n}) \\ &\qquad{}\times\prod_{i=2}^{n}u_{i}^{(-\alpha_{i}-1+\lambda _{i}\varepsilon)/p_{i}} \,du_{2}\cdots\,du_{n} \Biggr). \end{aligned}$$

It follows from (1), (6), and (7) that

8 $$\begin{aligned} &\int_{1}^{+\infty}x_{1}^{-1-\lambda_{1}c+\lambda_{1}\varepsilon}\,dx_{1} \\ &\qquad{}\times \Biggl( \int_{1}^{+\infty}\cdots \int_{1}^{+\infty }K(1,u_{2}, \ldots,u_{n})\prod_{i=2}^{n}u_{i}^{(-\alpha_{i}-1+\lambda _{i}\varepsilon)/p_{i}} \,du_{2}\cdots\,du_{n} \Biggr) \\ &\quad \leq\frac{M}{\varepsilon}\prod_{i=1}^{n} \biggl( \frac{1}{-\lambda _{i}{}} \biggr) ^{1/p_{i}}. \end{aligned}$$

Since $-1-\lambda_{1}c+\lambda_{1}\varepsilon>-1$, $\int_{1}^{+\infty }x_{1}^{-1-\lambda_{1}c+\lambda_{1}\varepsilon}\,dx_{1}$ diverges to +∞. Thus it is a contradiction to the above inequality. That is, it does not hold for $c>0$.

If $c<0$, for $0<\varepsilon<-c$, take

$$f_{i}(x_{i})= \textstyle\begin{cases} x{}_{i}^{(-\alpha_{i}-1-\lambda_{i}\varepsilon)/p_{i}}, & 0< x_{i}\leq1, \\ 0, & x_{i}>1,\end{cases} $$

where $i=1,\ldots,n$. Similarly, one can get

9 $$\begin{aligned} &\int_{0}^{1}x_{1}^{-1-\lambda_{1}c-\lambda_{1}\varepsilon} \, dx_{1} \Biggl( \int_{0}^{1}\cdots \int_{0}^{1}K(1,u_{2},\ldots ,u_{n}) \\ &\qquad{}\times\prod_{i=2}^{n}u_{i}^{(-\alpha_{i}-1-\lambda _{i}\varepsilon)/p_{i}} \,du_{2}\cdots\,du_{n} \Biggr) \\ &\quad \leq\frac{M}{\varepsilon}\prod_{i=1}^{n} \biggl( \frac{1}{-\lambda _{i}{}} \biggr) ^{1/p_{i}}. \end{aligned}$$

Since $-1-\lambda_{1}c- \lambda_{1}\varepsilon<-1$, $\int_{0}^{1}x_{1}^{-1-\lambda_{1}c-\lambda_{1}\varepsilon}\, {d}x_{1}$ diverges to +∞, which also contradicts the above inequality. It does not hold for $c<0$.

To sum up, we also get $c=0$ for $\lambda_{i}<0$ $(i=1,\ldots,n)$.

(ii) Suppose that (1) holds. If the constant factor $\inf M\neq\frac{W_{1}}{|\lambda_{1}|} \prod_{i=1}^{n}|\lambda_{i}|^{1/p_{i}}$, then there exists a constant $M_{0}<\frac{W_{1}}{|\lambda_{1}|}\prod_{i=1}^{n}|\lambda_{i}|^{1/p_{i}}$ such that

$$\int_{\mathbf{R}_{+}^{n}}K(x_{1},\ldots,x_{n})\prod _{i=1}^{n}f_{i}(x_{i})\,dx_{1}\cdots\,dx_{n}\leq M_{0} \prod_{i=1}^{n} \Vert f_{i} \Vert _{p_{i},\alpha_{i}}. $$

For sufficiently small $\varepsilon>0$ and $\delta>0$, take

$$f_{1}(x_{1})= \textstyle\begin{cases} x{}_{1}^{(-\alpha_{1}-1-|\lambda_{1}|\varepsilon)/p_{1}}, & x_{1}\geq1, \\ 0, & 0< x_{1}< 1.\end{cases} $$

For $i=2,3,\ldots,n$, take

$$f_{i}(x_{i})= \textstyle\begin{cases} x{}_{i}^{(-\alpha_{i}-1-|\lambda_{i}|\varepsilon)/p_{i}}, & x_{i}\geq \delta, \\ 0, & 0< x_{i}< \delta.\end{cases} $$

Therefore,

10 $$\begin{aligned} &\prod_{i=1}^{n} \Vert f_{i} \Vert _{p_{i},\alpha_{i}} \\ &\quad= \biggl( \int_{1}^{+\infty }x{}_{1}^{-1- \vert \lambda_{1} \vert \varepsilon} \, dx_{1} \biggr) ^{1/p_{1}}\prod _{i=2}^{n} \biggl( \int_{\delta}^{+\infty }x_{i}^{1- \vert \lambda _{i} \vert \varepsilon} \, dx_{i} \biggr) ^{1/p_{i}} \\ &\quad= \biggl( \frac{1}{ \vert \lambda_{1} \vert \varepsilon} \biggr) ^{1/p_{1}}\prod _{i=2}^{n} \biggl( \frac{1}{ \vert \lambda_{i} \vert \varepsilon }\cdot \frac{1}{\delta^{ \vert \lambda_{i} \vert \varepsilon}} \biggr) ^{1/p_{i}} \\ &\quad= \frac{1}{\varepsilon}\prod_{i=1}^{n} \biggl( \frac{1}{ \vert \lambda_{i} \vert } \biggr) ^{1/p_{i}}\prod _{i=2}^{n} \biggl( \frac{1}{\delta^{ \vert \lambda _{i} \vert \varepsilon}} \biggr) ^{1/p_{i}}, \end{aligned}$$

11 $$\begin{aligned} &\int_{\mathbf{R}_{+}^{n}}K(x_{1},\ldots,x_{n})\prod _{i=1}^{n}f_{i}(x_{i})\,dx_{1}\cdots\,dx_{n} \\ &\quad= \int_{0}^{1}x_{1}^{-\frac{\alpha_{1}+1+|\lambda_{1}|\varepsilon }{p_{1}}} \Biggl( \int_{\delta}^{+\infty}\cdots \int_{\delta}^{+\infty }K(x_{1}, \ldots,x_{n}) \\ &\qquad{}\times\prod_{i=2}^{n}x_{i}^{-\frac{\alpha_{i}+1+|\lambda _{i}|\varepsilon}{p_{i}}} \,dx_{2}\cdots\,dx_{n} \Biggr) \, dx_{1} \\ &\quad= \int_{0}^{1}x{}_{1}^{\lambda\lambda_{1}-\frac{\alpha _{1}+1+|\lambda _{1}|\varepsilon}{p_{1}}} \Biggl( \int_{\delta}^{+\infty}\cdots \int_{\delta}^{+\infty}K \bigl(1,x_{1}^{-\lambda_{1}/\lambda _{2}}x_{2}, \ldots,x_{1}^{-\lambda_{1}/\lambda_{n}}x_{n} \bigr) \\ &\qquad{}\times\prod_{i=2}^{n}x_{i}^{-\frac{\alpha_{i}+1+|\lambda _{i}|\varepsilon}{p_{i}}} \,dx_{2}\cdots\,dx_{n} \Biggr) \, dx_{1} \\ &\quad= \int_{1}^{+\infty}x_{1}^{\lambda\lambda_{1}-\frac{\alpha _{1}+1+|\lambda_{1}|\varepsilon}{p_{1}}-\lambda_{1}\sum _{i=2}^{n}\frac{\alpha_{i}+1+|\lambda_{i}|\varepsilon}{\lambda_{i}p_{i}}+\sum _{i=2}^{n}\frac{\lambda_{1}}{\lambda_{i}}} \Biggl( \int_{\delta x_{1}^{-\lambda _{1}/\lambda_{2}}}^{+\infty}\cdots \int_{\delta x_{n}^{-\lambda _{1}/\lambda_{n}}}^{+\infty} \\ &\qquad{}\times K(1,u_{2},\ldots,u_{n})\prod _{i=2}^{n}u_{i}^{-\frac {\alpha _{i}+1+|\lambda_{i}|\varepsilon}{p_{i}}} \, du_{2}\cdots\,du_{n} \Biggr) \, dx_{1} \\ &\quad\geq \int_{1}^{+\infty}x_{1}^{\lambda_{1} ( \lambda-\sum _{i=1}^{n}\frac{\alpha_{i}+1}{\lambda_{i}p_{i}}+\sum_{i=1}^{n}\frac{1}{\lambda _{i}}-\frac{1}{\lambda_{1}}-\sum_{i=1}^{n}\frac{|\lambda_{i}|\varepsilon}{\lambda_{i}p_{i}} ) } \, dx_{1} \Biggl( \int_{\delta}^{+\infty }\cdots \int_{\delta}^{+\infty} \\ &\qquad{}\times K(1,u_{2},\ldots,u_{n})\prod _{i=2}^{n}u_{i}^{-\frac {\alpha _{i}+1+|\lambda_{i}|\varepsilon}{p_{i}}} \, du_{2}\cdots\,du_{n} \Biggr) \\ &\quad= \int_{1}^{+\infty}x_{1}^{-1- \vert \lambda_{1} \vert \varepsilon} \, dx_{1} \Biggl( \int_{\delta}^{+\infty}\cdots \int_{\delta}^{+\infty }K(1,u_{2}, \ldots,u_{n}) \times\prod_{i=2}^{n}u_{i}^{-\frac{\alpha_{i}+1+ \vert \lambda _{i} \vert \varepsilon}{p_{i}}} \,du_{2}\cdots\,du_{n} \Biggr) \\ &\quad= \frac{1}{ \vert \lambda_{1} \vert \varepsilon} \int_{\delta}^{+\infty}\cdots \int_{\delta}^{+\infty}K(1,u_{2}, \ldots,u_{n})\prod_{i=2}^{n}u_{i}^{- \frac{\alpha_{i}+1+ \vert \lambda_{i} \vert \varepsilon}{p_{i}}} \,du_{2}\cdots\,du_{n}. \end{aligned}$$

It follows from (1), (10), and (11) that

$$\begin{aligned} &\frac{1}{ \vert \lambda_{1} \vert } \int_{\delta}^{+\infty}\cdots \int_{\delta }^{+\infty}K(1,u_{2}, \ldots,u_{n})\prod_{i=2}^{n}u_{i}^{-\frac{\alpha _{i}+1+ \vert \lambda_{i} \vert \varepsilon}{p_{i}}} \,du_{2}\cdots\,du_{n} \\ &\quad \leq M_{0}\prod_{i=1}^{n} \biggl( \frac{1}{ \vert \lambda_{i} \vert } \biggr) ^{1/p_{i}}\prod _{i=2}^{n} \biggl( \frac{1}{\delta^{ \vert \lambda _{i} \vert \varepsilon}} \biggr) ^{1/p_{i}}. \end{aligned}$$

Consequently,

$$\frac{1}{ \vert \lambda_{1} \vert }\prod_{i=1}^{n} \vert \lambda_{i} \vert ^{1/p_{i}} \int_{\delta }^{+\infty}\cdots \int_{\delta}^{+\infty}K(1,u_{2},\ldots ,u_{n})\prod_{i=2}^{n}u_{i}^{-(\alpha_{i}+1)/p_{i}} \,du_{2}\cdots\,du_{n}\leq M_{0} $$

as $\varepsilon\rightarrow0^{+}$. And then let $\delta\rightarrow 0^{+}$, we eventually get

$$\begin{aligned} &\frac{W_{1}}{ \vert \lambda_{1} \vert }\prod_{i=1}^{n} \vert \lambda_{i} \vert ^{1/p_{i}} \\ &\quad= \frac{1}{ \vert \lambda_{1} \vert }\prod_{i=1}^{n} \vert \lambda_{i} \vert ^{1/p_{i}} \int_{ \mathbf{R}_{+}^{n-1}}K(1,u_{2},\ldots,u_{n})\prod _{i=2}^{n}u_{i}^{-(\alpha _{i}+1)/p_{i}} \,du_{2}\cdots\,du_{n} \\ &\quad \leq M_{0}, \end{aligned}$$

this is a contradiction. Hence $\inf M=\frac{W_{1}}{|\lambda_{1}|}\prod_{i=1}^{n}|\lambda_{i}|^{1/p_{i}}$, i.e., the constant factor $\frac{W_{1}}{|\lambda_{1}|}\prod_{i=1}^{n}|\lambda_{i}|^{1/p_{i}}$ is the best. □

## Applications

### Theorem 2

*Suppose that* $n\geq2,\sum_{i=1}^{n}\frac {1}{p_{i}}=1\ (p_{i}>1),\lambda_{i}>0$ (*or* $\lambda_{i}<0$), $f_{i}(x_{i})\in L_{p_{i}-1}^{p_{i}}(0,+\infty), i=1,\ldots,n$. *Then*

$$\int_{\mathbf{R}_{+}^{n}}\frac{\min\{x_{1}^{\lambda_{1}},\ldots ,x_{n}^{\lambda_{n}}\}}{\max\{x_{1}^{\lambda_{1}},\ldots ,x_{n}^{\lambda _{n}}\}}\prod_{i=1}^{n}f_{i}(x_{i}) \,dx_{1}\cdots\,dx_{n}\leq \Biggl( n!\prod _{i=1}^{n} \vert \lambda_{i} \vert ^{\frac{1}{p_{i}}-1} \Biggr) \prod_{i=1}^{n} \Vert f_{i} \Vert _{p_{i},p_{i}-1}, $$

*where the constant factor is the best*.

### Proof

Set $\alpha_{i}=p_{i}-1,\lambda=0$, then $\sum_{i=1}^{n} \frac{\alpha_{i}+1}{\lambda_{i}p_{i}}=\lambda+\sum_{i=1}^{n}\frac{1}{ \lambda_{i}}$. Take

$$K(x_{1},\ldots,x_{n})=\frac{\min\{x_{1}^{\lambda_{1}},\ldots ,x_{n}^{\lambda_{n}}\}}{\max\{x_{1}^{\lambda_{1}},\ldots ,x_{n}^{\lambda _{n}}\}}, $$

then $K(x_{1},\ldots,x_{n})$ is a quasi-homogeneous positive function with parameters $(\lambda,\lambda_{1},\ldots,\lambda_{n})$, and

$$\begin{aligned} W_{1} &= \int_{\mathbf{R}_{+}^{n-1}}K(1,u_{2},\ldots,u_{n})\prod _{i=2}^{n}u_{i}^{-(\alpha_{i}+1)/p_{i}} \,du_{2}\cdots\,du_{n} \\ &= \int_{\mathbf{R}_{+}^{n-1}}\frac{\min\{1,u_{2}^{\lambda_{2}},\ldots ,u_{n}^{\lambda_{n}}\}}{\max\{1,u_{2}^{\lambda_{2}},\ldots ,u_{n}^{\lambda_{n}}\}}\prod_{i=2}^{n}u_{i}^{-1} \,du_{2}\cdots\,du_{n} \\ &= \prod_{i=2}^{n}\frac{1}{ \vert \lambda_{i} \vert } \int_{\mathbf {R}_{+}^{n-1}}\frac{\min\{1,t_{2},\ldots,t_{n}\}}{\max\{1,t_{2},\ldots,t_{n}\}}\prod _{i=2}^{n}t_{i}^{-1} \, dt_{2}\cdots\,dt_{n}. \end{aligned}$$

In view of [1], we get

$$\int_{\mathbf{R}_{+}^{n-1}}\frac{\min\{1,t_{2},\ldots,t_{n}\}}{\max \{1,t_{2},\ldots,t_{n}\}}\prod_{i=2}^{n}t_{i}^{-1} \,dt_{2}\cdots\,dt_{n}=n!, $$

it follows that

$$\frac{W_{1}}{ \vert \lambda_{1} \vert }\prod_{i=1}^{n} \vert \lambda_{i} \vert ^{\frac {1}{p_{i}}}=n!\frac{1}{ \vert \lambda_{1} \vert } \prod _{i=2}^{n} \vert \lambda _{i} \vert ^{-1}\prod_{i=1}^{n} \vert \lambda_{i} \vert ^{\frac{1}{p_{i}}}=n!\prod _{i=1}^{n} \vert \lambda_{i} \vert ^{\frac{1}{p_{i}}-1}. $$

According to Theorem 1, we know that Theorem 2 holds. □

### Theorem 3

*Suppose that* $n\geq2,\sum_{i=1}^{n}\frac {1}{p_{i}}=1\ (p_{i}>1),a>0,\lambda_{i}>0$, $\alpha_{i}\in\mathbf {R},p_{i}>1+\alpha _{i}$. *Then*

(i) *the inequality*
  12 $$ \int_{\mathbf{R}_{+}^{n}}\frac{1}{ ( x_{1}^{\lambda _{1}}+x_{2}^{\lambda _{2}}+\cdots+x_{n}^{\lambda_{n}} ) ^{a}}\prod_{i=1}^{n}x_{i}^{-\frac{\alpha_{i}+1}{p_{i}}} \,dx_{1}\cdots\,dx_{n}\leq M\prod _{i=1}^{n} \Vert f_{i} \Vert _{p_{i},\alpha_{i}} $$
  *holds for some constant* $M>0$ *if and only if* $\sum_{i=1}^{n}\frac{\alpha _{i}+1}{\lambda_{i}p_{i}}=-a+\sum_{i=1}^{n}\frac{1}{\lambda_{i}}$.
(ii) *if* (12) *holds*, *then its best constant factor is*
  $$\inf M=\frac{1}{\Gamma(a)}\prod_{i=1}^{n} \lambda_{i}{}^{\frac {1}{p_{i}}-1}\prod_{i=1}^{n} \Gamma \biggl( \frac{1}{\lambda_{i}} \biggl( 1-\frac {\alpha _{i}+1}{p_{i}} \biggr) \biggr). $$

### Proof

Set $K(x_{1},\ldots,x_{n})=1/ ( x_{1}^{\lambda _{1}}+x_{2}^{\lambda_{2}}+\cdots+x_{n}^{\lambda_{n}} ) ^{a}$, then $K(x_{1},\ldots,x_{n})$ is a quasi-homogeneous positive funct+ion with parameters $(-a,\lambda_{1},\ldots,\lambda_{n})$. By Lemma 2,

$$\begin{aligned} W_{1} ={}& \int_{\mathbf{R}_{+}^{n-1}}\frac{1}{ ( 1+x_{2}^{\lambda _{2}}+\cdots+x_{n}^{\lambda_{n}} ) ^{a}}\prod_{i=2}^{n}x_{i}^{-\frac{\alpha_{i}+1}{p_{i}}} \,dx_{2}\cdots\,dx_{n} \\ = {}&\lim_{r\rightarrow+\infty} \int_{x_{i}>0,x_{2}^{\lambda_{2}}+\cdots +x_{n}^{\lambda_{n}}\leq r}\frac{1}{ \{ 1+r [ ( \frac {x_{1}}{r^{1/\lambda_{1}}} ) ^{\lambda_{1}}+\cdots+( \frac{x_{n}}{r^{1/\lambda_{n}}} ) ^{\lambda_{n}} ] \} ^{n}} \\ &{}\times\prod_{i=2}^{n}x_{i}^{ ( 1-\frac{\alpha _{i}+1}{p_{i}} ) -1} \,dx_{2}\cdots\,dx_{n} \\ ={}& \lim_{r\rightarrow+\infty}\frac{r^{\sum_{i=2}^{n}\frac{1}{\lambda _{i}}-\sum_{i=2}^{n}\frac{1}{\lambda_{i}} ( 1-\frac{\alpha _{i}+1}{p_{i}}) }\prod_{i=2}^{n}\Gamma( \frac{1}{\lambda_{i}} ( 1-\frac{\alpha_{i}+1}{p_{i}} ) ) }{\prod_{i=2}^{n}\lambda_{i}\Gamma ( \sum_{i=2}^{n}\frac{1}{\lambda_{i}} ( 1-\frac{\alpha _{i}+1}{p_{i}} ) ) } \\ &{}\times \int_{0}^{1}\frac{1}{ ( 1+rt ) ^{a}}t^{\sum _{i=2}^{n}\frac{1}{\lambda_{i}} ( 1-\frac{\alpha_{i}+1}{p_{i}} ) -1} \, dt \\ ={}& \lim_{r\rightarrow+\infty}\frac{\prod_{i=2}^{n}\Gamma( \frac {1}{\lambda_{i}} ( 1-\frac{\alpha_{i}+1}{p_{i}} ) ) }{\prod_{i=2}^{n}\lambda_{i}\Gamma( \sum_{i=2}^{n}\frac{1}{\lambda _{i}}( 1-\frac{\alpha_{i}+1}{p_{i}} ) ) } \int_{0}^{r}\frac {1}{( 1+u ) ^{a}}u^{\sum_{i=2}^{n}\frac{1}{\lambda_{i}} ( 1-\frac{\alpha_{i}+1}{p_{i}} ) -1} \, du \\ ={}& \frac{\prod_{i=2}^{n}\Gamma( \frac{1}{\lambda_{i}} ( 1-\frac{\alpha_{i}+1}{p_{i}} ) ) }{\prod_{i=2}^{n}\lambda_{i}\Gamma ( \sum_{i=2}^{n}\frac{1}{\lambda_{i}} ( 1-\frac{\alpha _{i}+1}{p_{i}} ) ) } \int_{0}^{+\infty}\frac{1}{ ( 1+u ) ^{a}}u^{\sum_{i=2}^{n}\frac{1}{\lambda_{i}} ( 1-\frac{\alpha _{i}+1}{p_{i}}) -1} \,du \\ ={}& \frac{\prod_{i=2}^{n}\Gamma( \frac{1}{\lambda_{i}} ( 1-\frac{\alpha_{i}+1}{p_{i}} ) ) }{\prod_{i=2}^{n}\lambda_{i}\Gamma ( \sum_{i=2}^{n}\frac{1}{\lambda_{i}} ( 1-\frac{\alpha _{i}+1}{p_{i}} ) ) } \\ &{}\times\frac{\Gamma( \sum_{i=2}^{n}\frac{1}{\lambda_{i}} ( 1-\frac{\alpha_{i}+1}{p_{i}} ) ) \Gamma( a-\sum_{i=2}^{n}\frac{1}{\lambda_{i}} ( 1-\frac{\alpha_{i}+1}{p_{i}} ) ) }{\Gamma( a ) } \\ = {}&\frac{1}{\Gamma( a ) }\prod_{i=2}^{n} \frac{1}{\lambda _{i}}\prod_{i=2}^{n} \Gamma \biggl( \frac{1}{\lambda_{i}} \biggl( 1-\frac{\alpha _{i}+1}{p_{i}} \biggr) \biggr) \Gamma \biggl( \frac{1}{\lambda_{1}} \biggl( 1-\frac{\alpha_{1}+1}{p_{1}} \biggr) \biggr) \\ ={}& \frac{1}{\Gamma( a ) }\prod_{i=2}^{n} \frac{1}{\lambda _{i}}\prod_{i=1}^{n} \Gamma \biggl( \frac{1}{\lambda_{i}} \biggl( 1-\frac{\alpha _{i}+1}{p_{i}} \biggr) \biggr). \end{aligned}$$

Based on this, we can obtain

$$\frac{W_{1}}{\lambda_{1}}\prod_{i=1}^{n} \lambda_{i}^{\frac{1}{p_{i}}}= \frac{1}{\Gamma( a ) }\prod _{i=1}^{n}\lambda_{i}^{\frac {1}{p_{i}}-1} \prod_{i=1}^{n}\Gamma \biggl( \frac{1}{\lambda_{i}} \biggl( 1-\frac {\alpha _{i}+1}{p_{i}} \biggr) \biggr). $$

According to Theorem 1, we know that Theorem 3 holds. □
